# RE-AIM applied to a primary care workforce training for rural providers and nurses: the Department of Veterans Affairs' Rural Women's Health Mini-Residency

**DOI:** 10.3389/frhs.2023.1205521

**Published:** 2023-11-03

**Authors:** Rachel E. Golden, Aimee M. Sanders, Susan M. Frayne

**Affiliations:** ^1^ United States Department of Veterans Affairs, HSR&D Center for Innovation to Implementation (Ci2i), VA Palo Alto Health Care System, Veterans Health Administration, Palo Alto, CA, United States; ^2^ US Department of Veterans Affairs, Office of Women’s Health, Washington, DC, United States; ^3^ Division of Primary Care and Population Health, Department of Medicine, Stanford University, Stanford, CA, United States

**Keywords:** RE-AIM, women’s health, workforce training, rural, Veterans, evaluation

## Abstract

**Introduction:**

Application of the Reach, Effectiveness, Adoption, Implementation, and Maintenance (RE-AIM) framework to evaluate workforce education and training programs targeting clinical health care staff has received relatively little attention. This paper aims to contribute to this area with RE-AIM findings from a women's health-focused workforce training program implemented by the U.S. Department of Veterans Affairs (VA). Over the past two decades, the rapid expansion of the women Veteran population in VA has necessitated a quick response to meet clinical demand. To address this health care need, the VA Offices of Rural Health (ORH) and Women's Health (OWH) partnered to deploy a primary care workforce development initiative for Rural Providers and Nurses—the Rural Women's Health Mini-Residency (Rural WH-MR)—to train VA clinicians in rural locations in skills for the care of women Veterans. Here we assess the applicability of RE-AIM as an evaluation framework in this context.

**Methods:**

We evaluated the Rural WH-MR, relying on a primarily quantitative approach, rooted in RE-AIM. It included longitudinal and cross-sectional measurements from multiple quantitative and qualitative data sources to develop selected metrics. Data collection instruments consisted of pre-, post-, and follow-up training surveys, course evaluations, existing VA databases, and implementation reports. We developed metrics for and assessed each RE-AIM component by combining data from multiple instruments and then triangulating findings.

**Results:**

Results from the Rural WH-MR program for fiscal years 2018–2020 indicate that RE-AIM provides an instructive evaluation framework for a rural workforce training program, particularly in eliciting clarity between measures of Reach vs. Adoption and focusing attention on both provider- and patient-level outcomes.

**Discussion:**

We describe evaluation metric development and barriers to and facilitators of utilizing RE-AIM as an evaluation framework for a provider- and nurse-facing intervention such as this workforce training program. We also reflect upon RE-AIM benefits for highlighting process and outcomes indicators of a training program's success and lessons learned for evaluating rural workforce development innovations. Several of our observations have implications for training and evaluation approaches in rural areas with more limited access to health care services.

## Introduction

Since 2008, the U.S. Department of Veterans Affairs (VA) has been growing its workforce of trained Women's Health Primary Care Providers (WH-PCPs) (1) via a national Women's Health Mini-Residency (WH-MR) initiative (2). This expanded workforce has been crucial for meeting the healthcare needs of the rapidly expanding population of women Veterans in VA (3). A quarter of women Veteran VA outpatients have a rural residence (4). Shortages of WH-PCPs in rural areas (5) impede rural women's access to WH-PCPs and thwart VA's goal that at least 85% of women Veterans be assigned to a WH-PCP (6) regardless of the site's rurality. VA's national WH-MR would seem to be a potential solution to these shortages, but rural staff face attendance barriers including larger travel distances to get to centralized trainings. This in turn takes more time away from clinical care, and rural staff may not have patient care coverage during absences.

To address these barriers, in fiscal year 2018 (FY 2018) VA implemented an adapted version of the WH-MR workforce training program, called the Rural WH-MR, described in detail elsewhere (5). By training VA rural primary care staff (teams of WH-PCPs and nurses), the Rural WH-MR strives to improve rural women Veterans’ access to teams equipped with the expertise necessary to provide high quality care. The multifold program goals include: (1) increasing the number of rural women Veterans receiving care from PCPs/nurses with women's health expertise, (2) increasing training participants' women's health expertise in order to improve the quality of rural women's health care, (3) expanding the workforce of PCPs/nurses with women's health expertise at rural sites to improve women's health care capacity, (4) consistently delivering a women's health training program that meets the needs of rural-based PCPs/nurses, and (5) promoting ongoing, local and virtual women's health training opportunities to sustain high levels of women's health services capacity in rural areas.

**Figure 1:**
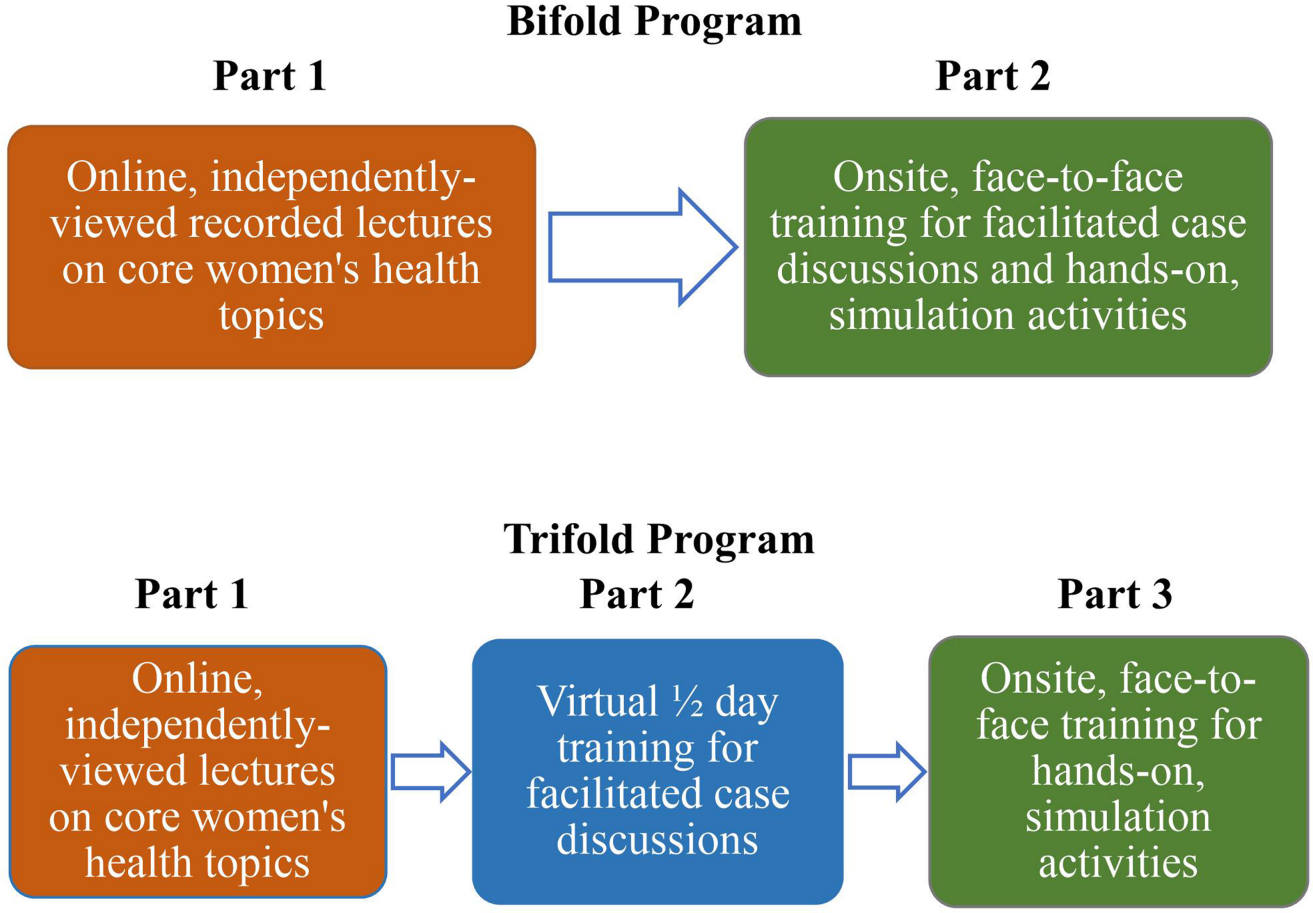
Rural Women's Health Mini-Residency bifold and trifold programs.

As shown in Figure 1, the “bifold” training program includes Part 1 (online recorded lectures completed independently) and Part 2 (a one-day, onsite, interactive training including facilitated case discussions, use of simulation equipment, and work with a live female model). A “trifold” adaptation, started in response to the Coronavirus (COVID-19) pandemic, divides Part 2 into two sessions: a half-day of case discussions facilitated virtually, followed by an abbreviated 1-day, hands-on training onsite, when pandemic conditions lift to the point that this becomes feasible. The data presented in this paper pertain to the bifold trainings delivered FY 2018-FY 2020, except where noted.

Evaluation of an innovative program adaptation like this is essential to inform ongoing program improvements and to determine the value of continuing to invest resources in the program. As a VA Office of Rural Health (ORH) funded program and in partnership with VA's Office of Women's Health (OWH), the Rural WH-MR training program developed an evaluation plan built on the Reach, Effectiveness, Adoption, Implementation, and Maintenance (RE-AIM) framework. RE-AIM has several advantages, including that it is widely used to evaluate behavioral health change interventions (7) and provides a means for cross-intervention comparisons and contrasts (8). RE-AIM has been used in diverse contexts over the past two decades (7–11) with increasing frequency. However, while other frameworks have been applied to the assessment of health care workforce training programs (12–15), RE-AIM has only recently been more widely used as a framework for such programs (16–22). The present paper seeks to expand the literature in this emerging area. Specifically, we present the Rural WH-MR as a case example to illustrate the application of RE-AIM for evaluation of a rural provider- and nurse-facing women's health training intervention. We describe metric development, reflect upon RE-AIM benefits for highlighting process and outcomes indicators, and discuss evaluation challenges and lessons learned.

## Materials and methods

### Data sources and data collection processes

This evaluation included aspects of both quantitative and qualitative program assessment. More details described elsewhere (5) delineate our data collection and analyses processes as well as our program's status as an operations project rather than research. VA's OWH made the determination of non-research. Therefore, the Rural WH-MR program did not need IRB approval. In this article we present quantitative methods and findings along with several supporting qualitative observations. Below, we briefly list data sources and data collection steps most of which are summarized in 2022 by Sanders et al (5). (See Table 1 for a compilation of RE-AIM component measures by their original data sources. Qualitative data collection instruments are available upon request).

**Table 1:** RE-AIM program components, measures, and key data sources.

Components: multi-fold program goals	Measures (program outcome levels):	Key data sources
Reach: increase # rural women Veterans receiving care from PCPs/nurses with WH expertise	**R1.** % rural women Veterans receiving care from trained PCPs (intermediate outcome)	• WHEI master ORH database^a^ • OWH tracking spreadsheets
Effectiveness: increase training participants’ WH expertise to improve the quality of rural WH care	**E1.** 5-point likert scale scores for PCP/nurse's self-reported comfort with training-related KAPS **E2.** % women Veterans provided with contraception (proximate and intermediate outcomes)	• OWH surveys • WHEI master ORH database^a^
Adoption: expand the workforce of PCPs/nurses with WH expertise at rural sites to improve WH care capacity	**A1.** % eligible sites participating **A2.** % rurality of participating sites **A3.** % availability of trained PCPs in rural sites (proximate outcomes)	• ORH rurality calculator • WHEI master ORH database^a^ • OWH tracking spreadsheets • Debriefing reports
Implementation: consistently deliver a WH training program that meets the needs of rural-based PCPs/nurses	**I1.** % Trainings with observer present **I2.** 5-point likert scale scores for PCP/nurse's view of usefulness of training activities **I3.** % PCPs/nurses satisfied with the training and program learning objectives (proximate & intermediate outcomes)	• OWH tracking spreadsheets • OWH surveys • ILEAD evaluations
Maintenance: promote ongoing, local & virtual WH training opportunities to sustain high levels of WH services capacity in rural areas	**M1.** % Rural WH-MR trainings attended by local leaders **M2**. # HCSs granted funding for subsequent local trainings **M3.** # online courses maintained & added (proximate & intermediate outcomes)	• OWH tracking Spreadsheets • Central OWH course-tracking system

WH, women's health; PCP, primary care provider; OWH, Office of Women's Health; VA, Department of Veterans Affairs; ORH, Offices of Rural Health; KAPS, knowledge, attitudes, practices, & skills; ILEAD, Institute for Learning, Education and Development; HCS, health care system.

^a^ This work draws upon VA's Women's Health Evaluation Initiative (WHEI) master ORH database (which pulls from multiple existing VA data sources), supplemented with files from VA's Corporate Data Warehouse, Primary Care Management Module (PCMM), VA Support Service Center capital assets (VSSC), and national Women's health Assessment of Workforce Capacity—primary care (WAWC-PC) data.

#### ORH rurality calculator

ORH provides an online calculator that OWH uses to determine site- and Health Care System (HCS)-specific rurality for program eligibility. VA uses the Rural-Urban Commuting Areas (RUCA) system to define rurality (23).

In addition, OWH tracks a variety of implementation and adoption processes with the following data sources:

#### OWH surveys

As described elsewhere (5), OWH administered electronic surveys (Qualtrics, Provo, UT) to PCP and nurse participants to assess their self-reported comfort with specific training-related knowledge, attitudes, practices, and skills (KAPS) at three time-points: (1) pre-training, (2) immediate post-training, and (3) six months post-training to track individual-level changes over time. (See Supplementary Appendix A for a summary of KAPS measures). The surveys also contained open-ended questions. For all trifold program trainings, OWH considered completion of the in-person part of the training as the index date for administering immediate and 6-month post-training surveys. The OWH Survey at the 6-month post-training mark also included a series of items about how useful case study discussions, simulation equipment, supply demonstration table and live female model activities were to participants' learning. Participants were also asked to rate the training's relevance to rural practice and share their thoughts about challenges and needs to care for women Veterans in a rural health care setting.

#### ILEAD evaluations

In parallel, VA's Institute for Learning, Education and Development (ILEAD) evaluations capture participant satisfaction, trainer performance, and course objectives achieved. Specifically, ILEAD evaluations are completed by participants within 30 days after training completion of the bifold program and after each part of the trifold program (due to separate program accreditations). These use 5-point Likert scales (strongly disagree, disagree, neither disagree nor agree, agree, strongly agree) to reflect participant satisfaction with respect to training applicability and delivery. Similarly, these evaluations report mean Likert scale scores showing whether participants feel that trainers met program-specific objectives. ILEAD evaluations also include open-ended items on these topics.

#### WHEI master ORH database

VA's Women's Health Evaluation Initiative (WHEI) compiles a longitudinal master ORH database of baseline and follow-up data (at the site-, PCP-, and patient-levels) that uses the training completion date as an index for measures taken pre-training, and 1- and 2-years post-training. The database taps into multiple existing national VA data sources via the WHEI Master Database supplemented with files from VA's Corporate Data Warehouse, Primary Care Management Module (PCMM), VA Support Service Center Capital Assets (VSSC), and national Women's Health Assessment of Workforce Capacity—Primary Care (WAWC-PC) data.

#### Debriefing reports

Additionally, the training site points of contact, site-observers, and contract trainers complete templated OWH post-training reports to reflect on implementation processes, while OWH also conducts a debriefing call with the site planning team to discuss implementation successes and challenges and to review post-training responsibilities (5).

#### OWH tracking spreadsheets

OWH had a site-observer (virtually or in-person) at many trainings given by new trainers and some given by repeat trainers. Data assessing the participating sites and training sessions were recorded in OWH Tracking Spreadsheets listing communications with sites and other site-specific details, including attendance of a site-observer (5). Further, all HCSs applying for and receiving funding to host local trainings were tracked by OWH in the spreadsheets, as was the percentage of trainings attended by local leaders.

#### OWH quality assurance reports

Site-observers provided immediate implementation feedback critiques to the trainers, and their quality assurance reports were used to assess trainers' adherence to training protocol and delivery to insure fidelity in training implementation (5).

#### Central OWH course-tracking system

To track the number of live and online courses offered in OWH's learning portfolio, OWH uses a central course-tracking system and lists available courses in VA internal communications to disseminate learning opportunities to clinical staff, also posting courses on the VA Training Management System.

#### VA contraception data

To assess contraceptive prescribing practices by PCPs, VA contraception pharmacy data were used in combination with a series of medical codes from outpatient utilization data to determine all types of contraception usage. Medical procedure and diagnostic codes came from a list compiled annually by Health and Human Services Office of Population Affairs (24), which uses all International Classification of Diseases (ICD-10), Current Procedural Terminology (CPT), and Health care Common Procedure Coding System (HCPCS) contraception provision codes.

All the data sources and collection processes described above were used to examine measures at multiple levels.

• **HCSs** examined include those that sent at least one PCP or nurse to the training.
• **Sites** refer to divisions within a HCS (such as the flagship VA Medical Center, or one of its satellite community-based VA outpatient clinics; not all sites within a participating HCS necessarily participate in the training).
• **Clinical staff** analyses examine PCPs (some of whom were already WH-PCPs at baseline) and nurses from participating sites.
• **Women Veteran** panel analyses examine women Veterans receiving care from a particular PCP.

### Operationalizing the RE-AIM components

In 2018, ORH's evaluation unit (25) provided evaluators with an information sheet (26) about RE-AIM with definitions and considerations to reflect the RE-AIM components. It defines **Reach** as “The absolute number, proportion and representativeness of individuals who are willing to participate in a given initiative, intervention or program”, and asks the evaluator to consider several issues, including whether the program reached the intended rural population. It also states that **Effectiveness** is “The impact of an intervention on important outcomes, including potential negative effects, quality of life, and economic outcomes”, followed with inquiries about how the intervention improves on current practice with respect to identified outcomes. The information sheet indicates that **Adoption** consists of “The absolute number, proportion and representativeness of settings and staff who actually initiate a program” and asks the evaluator several questions including whether the selected sites proved to be appropriate. Additionally, it states that **Implementation** specifically covers how closely the facilities and staff adhere to the various elements of an intervention's protocol, including consistency of delivery as intended and the time and cost of the intervention. The information sheet further notes that evaluators should account for whether the intervention was delivered with fidelity to the program's core elements and goals. Finally, it gives this definition of **Maintenance**: “The extent to which a program or policy becomes institutionalized or part of the routine organizational practices and policies” and asks the evaluator “What plans were developed to incorporate the intervention, so that it will be delivered over the long term?”

To apply RE-AIM to the Rural WH-MR, we first had to **operationalize** the RE-AIM components. We did this by carefully defining each component as it applied to the Rural WH-MR and selecting specific measures to assess program progress and impact. While our measures may not follow the RE-AIM framework exactly as applied to patient-facing interventions, we offer considerations for how we operationalized each component of the framework for our provider- and nurse-facing training program based on definitions provided by ORH.

#### Reach operationalized

We defined the **Reach** of the Rural WH-MR generally as the percentage of rural women Veterans receiving care from PCPs and nurses with women's health expertise, which in turn was defined as being (a) a WH-PCP and/or (b) completing the Rural WH-MR training program. We examined the effectiveness of the program's **Reach** according to increases in the following measure:

**R1.** The percentage of rural women Veterans on panels of WH-PCPs or PCP participants at program baseline and one- and two-years post-training.

To determine the percentage of rural women Veterans on panels of WH-PCPs or trained PCPs at program baseline and one- and two-years post-training, we considered each FY group of participants as a cohort. We then defined provider-specific training years such that T0 = training baseline date, T1 = one-year post-training follow-up date, and T2 = two-year post-training follow-up date. We took the first day of the training month for each provider as T0, which served as the index date; we added one and two years to create T1 and T2, respectively. We then used VA administrative data to link the records of all patients found at the training sites to their providers; we looked at those who were on the panels of trained PCPs at the specific timepoints, T0, T1, and T2. By selecting the rural women Veterans on PCPs' panels at each timepoint, we could determine the percentage point increase from T0 to T1 and T2.

#### Effectiveness operationalized

To show **Effectiveness**, we felt the program evaluation should reflect the program goal of increasing training participants' women's health expertise in order to improve the quality of rural women's health care. This led us to document two separate outcomes measures of Effectiveness:

**E1.** The changes over time in the Likert scale scores of participants’ self-reported comfort with training related KAPS.
**E2.** The percentage of women Veterans seen by trained PCPs provided with contraception by these participants at program baseline and one-year post-training.

To determine the PCPs' and nurses' changes in KAPS over time, we analyzed OWH survey data. Wilcoxon signed rank tests conducted in SAS (Version 9.4, NPAR1WAY procedure; pairwise deletion to address missing data) were used to analyze linked 5-point Likert scale data for all KAPS areas for pre- and post-training changes as well as pre- and 6-months post-training changes. Positive Likert scale scores indicated higher levels of comfort.

Subsequently, to determine contraception provision rates, we created provider-specific baseline and follow-up years with the baseline year, Y0, equal to the one-year period preceding T0, and Y1 equal to the one-year period starting with T0. Next, we identified all women Veterans seen by PCPs during each provider specific year (Y0 or Y1) by linking VA outpatient data to PCP identifying information for each visit with trained PCPs. To analyze changes from Y0 to Y1, we used a fixed effects logistic regression model with terms for year and dummy variables for stations to account for correlation. This allowed us to calculate the odds ratio of PCPs at 17 HCSs providing contraception at Y1 compared to Y0.

More specifically, pharmacy-based contraceptives include oral contraceptive pills, the depot medroxyprogesterone acetate injection, the vaginal ring, and the contraceptive patch. Other types of contraception from pharmacy data are not included in this measure because they are available over the counter (male and female condoms, spermicide), come from VA prosthetics (diaphragm, cervical cap, intrauterine device, implant) or are intended as back-up contraception (emergency contraception) and are not consistently reported in pharmacy data across sites. The WHEI list of medical codes is selected for the following types of contraception provision: any contraception, cervical cap, condom, diaphragm, implant, injectable, intrauterine device, oral contraception pills, patch, ring, spermicide, and sterilization; and does not include in the list of contraception provision any codes for contraception counseling, natural family planning, emergency contraception, abortion, pregnancy, live birth, non-live birth, or infecund.

#### Adoption operationalized

Prior to operationalizing adoption measures, we defined different units of analysis, counting these measures first by the fiscal year of each training cohort, and then for the program overall across all years cumulatively. For the Adoption measures, we defined and counted participating HCSs as those that hosted the Rural WH-MR and sent one or more PCPs to the training within given fiscal years. Similarly, participating sites were those VA Community-Based Outpatient Clinics (CBOCs) or VA Medical Centers (VAMCs) that sent at least one PCP to the training within that fiscal year. Training sessions counted for analysis comprised either the bifold one-day training date, or the second or third part of the trifold training dates. We also tracked the numbers of PCPs and nurses who partially and fully completed training (by fiscal year training cohort and cumulatively).

We measured the extent of program **Adoption** as:

**A1.** The percentage of eligible sites participating.
**A2.** The median percent rurality of participating sites.
**A3.** The percentage availability of WH-PCPs and trained PCPs in participating rural sites at program baseline and one-year post-training.

Sites eligible for the Rural WH-MR training are those primary care CBOCs and VAMCs where ≥50% of VA enrollees have a rural residence (as per the ORH rurality calculator based on RUCA). However, with ORH permission, non-rural primary care sites can also participate if they form part of a subset of sites with ≥50% rurality or they are sending participants to fill training spots that would otherwise go unfilled. The full denominator of eligible rural sites comes from the annual WAWC list of all VA primary care sites cross-referenced with the ORH rurality calculator. We included pilot sites in this measure.

To determine how well the set of the sites that adopted (participated in) the training aligned with the goal of reaching rural sites, we looked at participating sites' median rurality and the percentage that qualified officially as rural (per the ORH rurality calculator). We included pilot sites in this measure as well.

To be counted as a trained PCP, for bifold trainings, participants completed the one-day, in-person training session, but for trifold trainings, we tracked PCPs separately who partially vs. fully completed the training. We then examined the number of WH-PCPs before and after the trainings as well as participants who completed the training but may not have obtained WH-PCP status according to VA workforce data. However, since PCPs could qualify as WH-PCPs prior to the training, we reviewed the changes in percentages of WH-PCPs and/or training participants as an indicator of adoption. To assess the changes in availability of WH-PCPs at specific participating sites, we also relied on the annual WAWC counts of WH-PCPs at the sites in the year prior to and the year following the training to determine which participants were or became WH-PCPs after training.

#### Implementation operationalized

We defined program **Implementation** as consistently delivering a women's health training program that meets the needs of rural-based PCPs and Nurses. To do so, the Rural WH-MR needed to maintain fidelity to core program elements and cross-site consistency while allowing flexibility for site-specific tailoring. We monitored several measures to assess Implementation processes. These include:

**I1.** The percentage of trainings attended by site observers.
**I2.** Likert scale scores from participants regarding usefulness of training activities.
**I3.** The percentages of participants satisfied with the training and program learning objectives.

As noted above in the Data Sources and Collection Processes section, OWH had site-observers attend select trainings and complete quality assurance reports to critique trainers and reflect upon their fidelity to training implementation protocols. The total number of site-observers was monitored in the OWH Tracking Spreadsheets also described earlier.

We analyzed the quantitative OWH Survey 6-month post-training usefulness and relevance data with Wilcoxon signed ranks tests for participants' mean Likert scale scores (higher scores indicating more usefulness and relevance), while we also reviewed the qualitative data for commonly cited themes.

As noted earlier, ILEAD evaluations are completed by participants within 30 days after training completion of the bifold program and after each part of the trifold program. The 5-point Likert scales were used to assess participant satisfaction and to measure whether participants felt that trainers met program-specific objectives.

#### Maintenance operationalized

Finally, we defined **Maintenance** as promoting ongoing local and virtual women's health training opportunities to sustain high levels of women's health services capacity in rural areas. We used the following process measures for FY 2018-FY 2020 to assess effective program Maintenance:

**M1.** The percentage of the Rural WH-MR trainings attended by local leaders.
**M2.** The number of HCSs granted funding for subsequent local trainings.
**M3.** The number of live and online courses maintained and added to the OWH learning portfolio.

We postulate that local leaders who attended trainings are more likely to provide future local in-services, that additional funding of HCSs propagates the intervention, and that maintaining the courses online allows for rural sites to use the curriculum locally for their own in-services. We monitored local leader attendance via the OWH Tracking Spreadsheets described in the Data Sources and Collection Process section above. All HCSs applying for and receiving grant funding to host local trainings were tracked in a similar manner by OWH. To document the number of live and online courses offered in OWH's learning portfolio, OWH uses its central course-tracking system (also described above).

## Results

With the RE-AIM components operationalized and specific measures defined, we assessed the Rural WH-MR program's progress and impact. Here, we share results from FY 2018-FY 2020 showing that the RE-AIM approach successfully identified effective implementation and positive program performance.

### Reach results

**R1.** The percentage of rural women Veterans on panels of WH-PCPs or PCP participants at program baseline and one- and two-years post-training.

**Figure 2:**
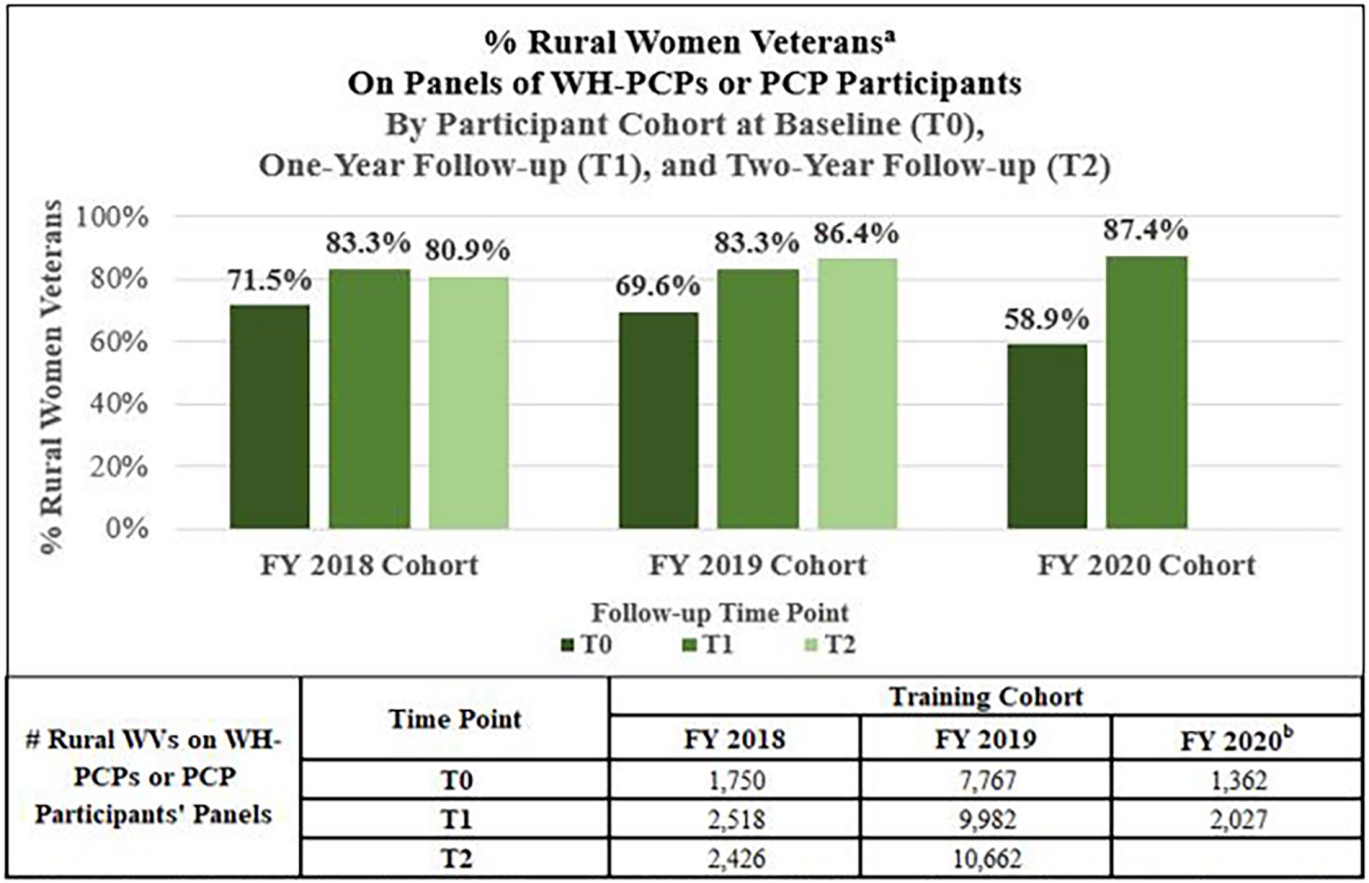
Among rural women Veterans at the participating health care systems percentage of rural women Veterans on Women's health primary care provider (WH-PCP) or primary care provider (PCP) participant panels. WH-PCP, Women's Health Primary Care Provider; PCP, Primary Care Provider; TO, Baseline year, TI, One-year follow-up; T2, Two-year follow-up; WVs, Women Veterans. ^a^Percentage of rural women Veterans on panels of WH-PCPs or PCP participants among all rural women Veterans at the participating Health Care Systems at baseline (T0), one-year follow-up (TI), and two-year follow-up (T2). ^b^Calculated without Spokane data due to Electronic Medical Record transition in 2020 leading to incomplete data for the Spokane Health Care System. Fewer Health Care Systems participated in FY20 due to COVID-19.

Using the described measures and methods, the percentage of rural women Veterans on trained PCPs' patient panels generally increased over time for all training cohorts. Figure 2 shows the percentages and absolute numbers of rural women Veteran primary care patients on panels of PCPs with women's health expertise. For FY 2018 HCSs, from T0 to T2, the absolute number of rural women Veterans on panels of PCPs with women's health expertise increased from 1,750 to 2,426 (an absolute increase of 676 rural women Veterans). Viewed as percentages, this translates to an increase from 71.5% to 80.9% (+9.4 percentage points) or a 38.6% relative increase in the percentage of rural women Veterans with access to providers with expanded women's health expertise. Similarly, among all participating sites from the selected HCSs in the FY 2019 and FY 2020 training cohorts, the percentages and absolute numbers of rural women Veterans on the panels of PCPs with women's health expertise increased from T0 to T2 as shown in Figure 2.

### Effectiveness results

**E1.** The changes over time in the Likert scale scores of participants’ self-reported comfort with training related KAPS.

Of *N* = 184 FY 2018–2020 PCP participants who completed the bifold program, none were lost to follow-up at the 1-month post-training time, and 74 (40%) had linked data available for both pre- and immediate post-training. Wilcoxon signed rank test analyses reveal significant improvements for all 22 PCP KAPS areas assessed (range of change +0.03 to +1.22, all *p*-values < 0.01) immediately post-training. For the 6-month post-training mark, 9 PCPs were lost to follow-up, with *N* = 175 PCPs remaining. As previously reported for the FY 2018 and FY 2019 PCP participant cohorts, 52 PCPs had linked data for pre- and 6-month post-training surveys. No new linked surveys were collected for the FY 2020 cohort. At 6-months post-training, none of the 22 KAPS means scores declined. Specifically, 18 increased significantly (range of change +0.25 to +0.88, *p* < .03 for each), while four did not show statically significant improvement.

Likewise, of the *N* = 327 FY 2018–2020 nurse participants who completed the bifold program, none were lost to follow-up at the 1-month post-training time, and 186 (57%) had linked data available for both pre- and immediate post-training. Of the 17 nurse KAPS areas assessed, Wilcoxon signed rank test analyses indicate significant improvements for all of them (range of change + 0.51 to +1.23, all *p*-values < 0.01) among those nurses immediately post-training. As previously reported for the FY 2018 and FY 2019 nurse participant cohorts, 93 nurses had linked data for pre- and 6-month post-training surveys. No new linked surveys were collected for the FY 2020 cohort. At 6-months post-training all 17 nurse KAPS areas assessed increased significantly (range of change +0.39 to +0.84, *p* < .01 for each).

**E2.** The percentage of women Veterans seen by trained PCPs provided with contraception by these participants at program baseline and one-year post-training.

As shown in Table 2, for women Veterans under age 50 who had at least one outpatient encounter with a FY 2019 PCP participant during the one-year, provider-specific ascertainment periods, the HCS median percentage of those women Veterans who were provided contraception by the trained PCPs increased from 3.9% at baseline to 6.3% at follow-up. According to the logistic regression analyses, the odds of trained PCPs providing contraception in the follow up year were significantly higher than in the baseline year, with an odds ratio of 1.32 [95% Confidence Interval (1.12, 1.56), *p* = 0.0009].

**Table 2:** Participants’ provision of contraception to the 18–49-year-old women Veterans whom they saw pre-training vs. post-training^a.^

Among women Veterans ages 18–49 who saw a PCP participant during Y0 or Y1, percent who received contraception from the PCP: HCS median rate^a^ (range) *N* = 17 HCS		Δ Y1–Y0 (absolute change in percentage points)	Δ (Y1–Y0)/Y0 (relative percent change)	OR (CI), *p*-value for OR^b^
Y0	Y1			
3.9% (0%–12.0%)	6.3% (2.8%–13.9%)	2.4	61.5%	1.32 (1.12, 1.56), *p* = 0.0009

OR—Odds Ratio; CI—95%, Confidence Interval.

^a^ The denominators for the baseline (Y0) and follow-up (Y1) rates examined here are women Veterans ages 18–49 years old who had at least one visit with an FY 2019 PCP participant during the provider-specific Y0 or Y1, respectively. The numerator is the subset of those women who received contraception from a trained PCP during the corresponding Y0 or Y1 period, based on medical codes and VA pharmacy data files. At each Health Care System (HCS), the number of women in the numerator across trained providers is divided by the number of women in the denominator across providers, and then the across-HCS median percentage (and range) is presented.

^b^ *p*-value from fixed effects logistic regression models.

### Adoption results

**Figure 3:**
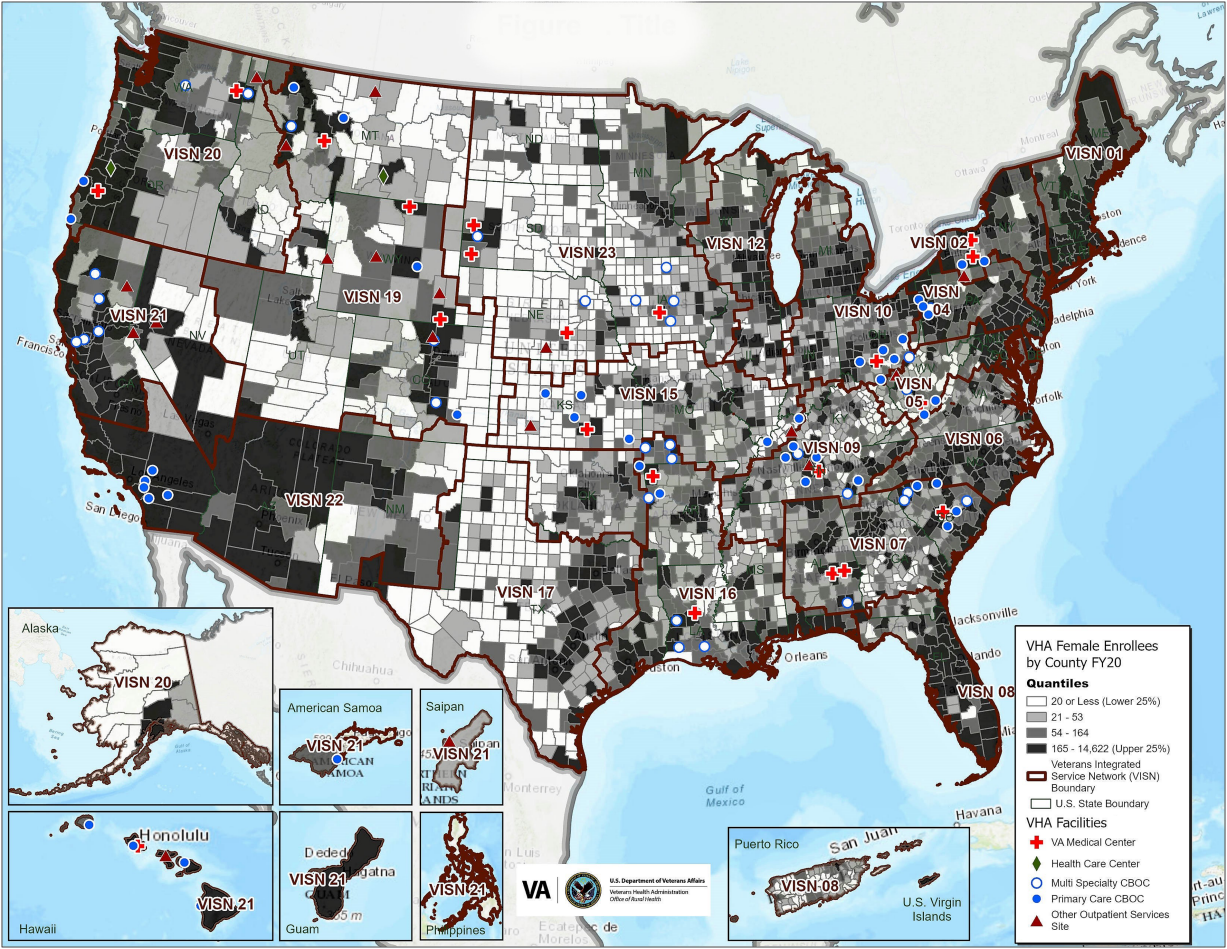
Map—Rural Women's Health Mini-Residency participating sites (substations) including pilot and fiscal years 2018–2020. Grey shades of the map's background show fiscal year 2020 rural female VA enrollees by county (darker shades indicating higher numbers of rural women Veterans) and the colored symbols indicate practice sites of primary care providers and/or nurses who participated in the training whether or not the sites met the rurality threshold (≥50% rurality per the Office of Rural Health rurality calculator). VHA, Veterans Health Administration; FY, fiscal year; VISN, Veterans integrated service network; CBOC, community based outpatient clinic.

Table 3 delineates program process measures by fiscal year, training locations, events, and encounters. The map shown in Figure 3 shows the “footprint” of the Rural WH-MR inclusive of pilots and all FY 2018-FY 2020 sites that sent PCPs and/or nurses to bifold and trifold trainings and indicates the geographic distribution of all participating sites whether or not they met the rurality threshold. As seen, by the end of FY 2020, the Rural WH-MR had gone as far east as New York, west as the Pacific Islands, north as Montana, south to Louisiana and many places in between.

**A1.** The percentage of eligible sites participating.

**Table 3:** Total^a^ health care systems (HCSs) participating, training sessions held, sites (substations) represented, and participants, by fiscal year (FY).

FY	Session type^b^	#HCS		#Training sessions	#Sites		#PCPs trained	#Nurses trained
		PCPs and/or nurses	Nurses only		PCPs and/or nurses	Nurses only		
Pilots	Bifold	2	0	4	8	0	14	23
FY 2018	Bifold	4	0	13	23	4	32	54
FY 2019	Bifold	17	0	39	85	12	135	243
FY 2020	Bifold	3	0	6	10	2	17	30
	Trifold	2	0	6	10	2	18	38
Totals (unique entities or individuals)		**25**	**0**	**68**	**126**	**20**	**216**	**388**

FY, Fiscal Year; PCP, Primary Care Provider.

^a^ Health care systems (HCSs), participating sites (i.e., within the participating HCSs, those sub-stations that sent at least one PCP or nurse to the training in the specified fiscal year), and primary care providers (PCPs)/nurses may appear more than once in separate rows for instances in which they returned for further training in subsequent years; therefore, totals of unique entities or unique individuals may be less than the sum of the year counts.

^b^ Bifold session refers to the rural Women's health Mini-Residency's usual 2-part program (online recorded lectures and 1-day onsite training); trifold session refers to the rural Women's health Mini-Residency's adapted 3-part program used during the coronavirus pandemic (online recorded lectures, virtual case study discussions, and abbreviated onsite training when conditions permit).

At the end of FY 2020, of the *N* = 1,055 primary care sites found in the ORH Rurality Calculator FY 2020 list of sites, 510 (48.3%) primary care sites were ≥50% rural and therefore eligible to participate in the Rural WH-MR on the basis of rurality. Of those 510 eligible primary care rural sites, 65 (12.7%) had sent PCPs to the Rural WH-MR during pilot or FY 2018-FY 2020 trainings.

On top of those 65 eligible rural sites, an additional 41 other sites sent PCPs. Among them, one was rural (but had unknown primary care status, i.e., missing from WAWC FY 2020 which was the source of sites' primary care status), 39 did not meet the rurality threshold in the ORH Rurality Calculator but had received permission from ORH to send participants to the Rural WH-MR, and one had unknown rural status (a mobile clinic, missing from the ORH Rurality Calculator).

**A2.** The median percent rurality of participating sites.

A total of 106 unique sites sent PCPs to a pilot or FY 2018-FY 2020 training. (Twenty additional sites sent nurses but not PCPs to training during those years.) Excluding the one site missing from ORH's Rurality Calculator, the participating 105 sites had a median rurality of 69.5% (range: 0%–100% of all Veterans enrolled at the site had a rural residence). Specifically, 66 participating sites (62.9%) were ≥50% rural, and 39 participating sites (37.1%) did not meet the rurality threshold.

**A3.** The percentage availability of WH-PCPs and trained PCPs in participating rural sites at program baseline and one-year post-training.

The availability of WH-PCPs and trained PCPs improved from baseline to one-year follow-up for the FY 2018-FY 2020 cohorts as assessed by WHEI using the annual WAWC surveys to determine WH-PCP status of participants prior and subsequent to training. Among the *N* = 202 FY 2018-FY 2020 PCP participants (includes trifold participants in 2020 who could not complete the final phase of the training until the following fiscal year due to the Coronavirus pandemic), 87 (43%) were WH-PCPs before their training. At the one-year follow-up point, among the original 202 PCP participants:

• 168 (83.2%) were WH-PCPs.
• 17 (8.4%) PCPs had left VA or changed positions.
• 6 (3.0%) anticipated completing the training in the following fiscal year due to delays imposed by the Coronavirus pandemic.
• 10 (5.0%) were either float providers or home-based PCPs functioning as WH-PCPs.
• Only 1 (0.5%) decided not to become a WH-PCP.

### Implementation results

**I1.** The percentage of trainings attended by site observers

In FY 2018, 54% (7 out of 13) of trainings were attended by a site observer. In FY 2019 and FY 2020, 44% and 50% (17 out of 39, and 6 out of 12) of trainings had site observers, respectively. A decreased frequency reflects a year in which more trainings were delivered by previously observed training teams, seen in the past without noted deficiencies and therefore not needing close observation. In fact, among trainings from FY 2018-FY 2020 that had at least one new training team member, 54% (7 out of 13) received observation from an OWH representative or designee.

**I2.** Likert scale scores from participants regarding usefulness of training activities.

Using a scale in which 1 = poor and 5 = excellent, PCPs (*N* = 106 responding) from FY 2018-FY 2020 rated case studies, simulation equipment, MammaCare®, live female model (Gynecologic Teaching Associate, GTA) experience, and supply demonstration as 4.49–4.69. In the words of a FY 2019 PCP post-training: “*It is really wonderful to have access to a GTA. I always feel like I am able to improve my skills with constructive feedback”.* Nurses (*N* = 251 responding) from these cohorts rated case studies, exam set-up activity, gynecologic procedure videos, live female model experience, and supply demonstration as 4.71–4.82. A nurse from the FY 2019 cohort reported post-training: “*The case studies were very useful in learning how to interact with a patient regarding sensitive subjects as well as line of questioning that will help the provider give a more clear course of care*”*.* Finally, FY 2018-FY 2020 OWH survey data demonstrated strength of the training's relevance to rural practice, with ratings of 4.50 among PCPs (*N* = 110 responding) and 4.71 among nurses (*N* = 256 responding).

**I3.** The percentages of participants satisfied with the training and program learning objectives.

Response rates for the Rural WH-MR ILEAD Evaluations for FY 2018-FY 2020 were 94.0% (PCPs, *N* = 173) and 89.3% (nurses, *N* = 292). Cumulative FY 2018-FY 2020 data showed high participant satisfaction (PCPs: 94.3% and nurses: 95.9% across respective 9-question and 8-question, 5-point Likert scale composite measures). Similarly, 93.4% of PCPs indicated satisfaction across 10 PCP program-specific objectives, while 94.9% of nurses showed satisfaction across 8 nurse program-specific objectives.

### Maintenance results

**M1.** The percentage of Rural WH-MR Trainings attended by local leaders.

Local women's health leaders attended 100% of trainings once an expectation for their attendance became policy in FY 2019, at which point it was clear to OWH that trainings ran more smoothly with the leaders present. Additionally, participants found benefit when local women's health leaders attended the Rural WH-MR as noted by this participant's evaluation comment, “*It was also helpful that local leadership was present to offer immediate feedback to topics being presented*”*.*

**M2.** The number of HCSs granted funding for subsequent additional local trainings.

Of the 25 unique HCSs that participated in the Rural WH-MR in a pilot or during FY 2018-FY 2020, four (4) received additional funding from OWH, training materials, and program support to host their own local women's health trainings in subsequent years (2 HCSs were funded multiple times). Additionally, at least 11 FY 2018-FY 2020 Rural WH-MR-participating HCSs also participated in regional-wide women's health trainings supported by OWH in the years after having the Rural WH-MR.

**M3.** The number of Online courses maintained & added to the OWH learning portfolio.

By FY 2020, there were more than 40 hours of online training content available for local trainers in the OWH online library including on-demand courses, slide decks, breast and pelvic exam instructional videos, simulation equipment instructions, and other supporting training-related materials. OWH ensures training content is regularly reviewed and updated to reflect the most up-to-date, evidence-based medical guidance available. Additionally, over 100 women's health clinically oriented online, on-demand courses were widely available for VA staff to take advantage of for their ongoing learning needs.

### Evaluation challenges and benefits of RE-AIM, and lessons learned for evaluating training programs in rural areas

Sanders et al. (2022) (5) delineate program adoption and implementation barriers for the Rural WH-MR as well as strategies for addressing those barriers. In the current article's discussion, we examine some RE-AIM program evaluation barriers, benefits, and lessons learned for assessing training programs in rural areas. In short, given the challenge of applying RE-AIM to a workforce training, we found that to conduct a robust program evaluation, host training agencies should:

• Enlist evaluation expertise prior to program onset to ensure consistent and accurate use of RE-AIM for the program's duration.
• Ensure that flexibility is built into the training's structure so that implementation and evaluation can weather unforeseen events (e.g., COVID-19) while remaining aligned with RE-AIM.
• Distinguish the difference between Reach and Adoption measures early in the evaluation to devise separate measures reflecting these RE-AIM components differently.

The latter point emphasizes one of the benefits of RE-AIM for evaluation–it helps to highlight opportunities to measure patient vs. clinician program outcomes.

## Discussion

Utilizing RE-AIM to guide the Rural WH-MR's evaluation plan enabled us to take a systematic approach to developing our program's metrics and ultimately facilitated effective data collection for evaluation of this provider- and nurse-facing workforce training initiative. Along the way, the application of RE-AIM to our program evaluation also brought to light some challenges. Initially, our evaluation team defined our intended populations as both (1) PCPs and nurses serving rural women Veterans, and (2) rural women Veteran patients. As such we sought to measure the percentage of eligible PCPs and nurses “reached” (trained by the Rural WH-MR) as well as the percentage of rural women Veterans reached (on the panels of designated WH-PCPs). Both measures introduced challenges. For the former, we struggled with the selection of an appropriate denominator for PCPs and nurses, as sites frequently replaced selected participants due to scheduling conflicts. For the latter, since PCPs could be WH-PCPs prior to the training and not all trained PCPs became designated WH-PCPs, we needed two outcome measures, one reflecting rural women Veterans on WH-PCP panels and the other accounting for rural women Veterans on WH-PCP and/or participant panels. After further discussion with ORH about operationalization of Reach and Adoption, we reframed Reach in terms of patient-level outcomes and Adoption in terms of health care staff-level and health care facilities-level outcomes. We hope that our experience with applying RE-AIM in a rural setting and identifying rural-specific measures (e.g., rurality of sites) will benefit those applying RE-AIM to rural settings, and that our experience with this application of RE-AIM will expand a relatively limited existing literature on the use of RE-AIM for PCP/nurse training initiatives.

When tracking the numbers and percentages of rural women Veterans on panels of PCPs with women's health expertise for each Rural WH-MR training cohort over time, we see general improvement longitudinally through increasing rates of patient coverage. The trend points to program success, although without a comparison group, we cannot conclude definitively that the program increased the percentages of rural women Veterans on panels of WH-PCPs and/or participants. Future evaluation activities may involve creating an intervention and a comparison group to allow for difference-in-difference analyses.

We have seen in the literature that RE-AIM measures do not always align across different evaluations, with the evaluators citing different types of measures as indicators of varying RE-AIM components. Holtrop et al. (2021) (11) outline common misunderstandings in the application of RE-AIM and advise use of patient-level outcomes for the Effectiveness component of RE-AIM. In our application of RE-AIM, in which the recipients of the intervention are clinical staff and not patients, we account for the focus on the provider- and nurse-level training as a proximate measure of training Effectiveness via changes in KAPS (a trainee-level measure, widely used in evaluations of training interventions) (27–30). Indeed, improvements in KAPS are a necessary antecedent but not often sufficient alone to create demonstrable improvements in patient outcomes. Researchers reviewing studies of clinician-facing interventions have found that a small percentage of studies are designed in such a way as to be able to demonstrate a direct impact on patient health. Among the strengths of our primary evaluation is that in addition to the trainee-level Effectiveness measure directly attributable to the training, we also examined a more distal, patient-level effectiveness measure (receipt of contraception).

Specifically, in 2020, we began to examine women's health outcome indicators that we could measure to reflect the program Effectiveness. We considered as possibilities the percentages of rural women Veterans receiving appropriate: (1) contraception, (2) cancer screenings, and (3) cancer screening results follow-up, among others. To determine clinical appropriateness, all measures required complex algorithms, but in the case of contraception, we felt that given the apparent needs around contraception among women Veterans generally (31) we could safely assume that increases in provision of contraception to women Veterans of reproductive age likely indicated improvements in providers' women's health care aptitude. Medical codes commonly indicate when PCPs have provided contraception procedures or prescriptions, and since the training focuses on the providers and nurses, we postulated that PCP contraception provision increases among women Veterans would likely reflect a positive program impact.

For our contraception provision rates, we see that, although the rates before and after the training are small, the large relative percentage increase in contraception provision is clinically meaningful and statistically significant. However, future work should examine contraception provision in intervention groups and appropriately selected comparison groups.

Regarding program Adoption, as our results show approximately 13% of eligible sites sent PCPs and/or nurses to the Rural WH-MR for pilot or FY 2018-FY 2020 trainings, leaving many eligible sites yet to receive training. Nevertheless, given that the program accomplished this participation rate in essentially 3 years (since in FY 2017 only one pilot HCS participated and in FY 2020, COVID-19 hindered in-person training sessions), the Rural WH-MR has shown high program Adoption in a relatively short time.

Further, with respect to program Adoption, the median rurality of participating sites was just under 70%. It would have been even higher except that some less rural sites were accommodated on a case-by-case basis to optimize use of available training spaces. Furthermore, although the majority of participating sites were rural, the less rural sites that sent PCPs and/or nurses still serve rural women Veterans among their patient population, thus allowing program benefits to disseminate. These Adoption metrics all suggest that the site selection process effectively identified rural sites appropriate for the program goal of increasing access for rural women Veterans to PCPs and nurses with women's health expertise.

Among PCP participants with pre/post data, the percentage serving in a designated WH-PCP role nearly doubled from pre-training to post-training, such that 83% were designated WH-PCPs at follow-up, an additional 5% essentially functioned as WH-PCPs, and 3% could not finish until the following year due to the Coronavirus pandemic. All, even those without the formal WH-PCP designation, were now better positioned to serve rural women Veterans with increased women's health expertise due to their exposure to up-to-date women's health training activities and content.

During the FY 2018-FY 2020 trainings, the Rural WH-MR was well implemented throughout the country as per our metrics. For example, we report that over one half of trainings in which there was at least one new training team member were monitored by OWH representatives or designees ensuring high program implementation fidelity and quality. Other measures used to evaluate the Rural WH-MR's Implementation such as usefulness of the training activities, the training's relevance to rural practice, and participant satisfaction, reflect a well-targeted curriculum and appropriate set of skills development exercises.

Lastly, RE-AIM asks whether programs reflect widespread Maintenance as well as Reach, Adoption, and Implementation. While we acknowledge that our Maintenance measures may not follow the RE-AIM framework in the traditional sense as used for patient-facing interventions, we offer considerations for how we defined Maintenance for our Rural WH-MR provider- and nurse-facing training. An Effective program should continue to thrive after the initial efforts, so we looked for indicators that the Rural WH-MR could be sustained. These measures became more evident during program implementation, as we realized which types of efforts contribute to ongoing trainings of rural PCPs and nurses. For example, after engaging with the Rural WH-MR, at least 15 HCSs either hosted their own local women's health trainings or participated in regional trainings, both of which provide opportunities for participants to use their experience to disseminate more training information to additional staff.

With 100% of in-person trainings attended by local leaders, Rural WH-MR training sessions became more tailored to local sites because these leaders could speak to site-specific concerns. This in turn improved the quality of the trainings and the likelihood of maintaining aspects of the training over longer time periods. As one PCP stated after training in FY 2020: “*Very helpful to attend to update my own knowledge. In addition, valuable for me as the physician team lead to join in the experience to be able to continue to teach/mentor my colleagues*”*.*

To further support ongoing dissemination of training after participating in the Rural WH-MR, we note that the number of online courses maintained and added to the OWH online library grew impressively in response to the demand. Making these materials available and maintaining them so that they contain the most recent recommendations requires ongoing curation by OWH, but the benefits of the expansive offerings include easy accessibility and allowing for further propagation of women's health knowledge and skills development activities within VA.

As a result of all program metrics—defined within the RE-AIM framework—showing positive program progress through FY 2020, ORH Leadership in FY 2021 committed to continue the OWH-ORH partnership to support the Rural WH-MR for 5 additional years (FY 2022-FY 2026). As discussed, many eligible rural sites have yet to benefit from this program, and the additional funds will allow for ongoing program Maintenance, while in the meantime, OWH explores ways in which the Rural WH-MR can be partially absorbed and implemented within the grander national WH-MR training program structure.

Other education-focused initiatives for health professionals may build upon our experience using RE-AIM as an evaluation framework for a workforce training program. The framework helped to distinguish provider- and patient-level program outcomes of interest and created the context for understanding other aspects of the program assessment.

With respect to the overall evaluation challenges and benefits of RE-AIM, and lessons learned for evaluating training programs in rural areas, OWH confirmed that enlisting evaluation expertise from the start has substantially enhanced all stages of program development and implementation. This aligns with the principle that the evaluation cycle should occur in concert with all stages of program design and rollout (32, 33). In fact, program uptake required ongoing, strong partnerships to help design the multifaceted evaluation processes, analyze data, and monitor outcomes from restricted databases. While the education and training developers had expertise in program implementation, WHEI and ILEAD specialize in evaluation, allowing for a robust program assessment in accordance with ORH program requirements.

Additionally, OWH learned that ensuring that flexibility is built into the training's structure can help both the program implementation and evaluation to weather unforeseen events such as COVID-19. The pandemic, in fact, proved the biggest obstacle to program implementation and evaluation by precluding numerous in-person trainings until they were deemed safe to implement. To overcome the obstacle of the COVID-19 pandemic, OWH offered the trifold program in addition to the bifold program. This enabled HCSs to continue a path towards completing the training in full, though extended over time, by participating in the in-person training when COVID-19-safe conditions arose locally.

RE-AIM has only recently been more widely suggested as a framework for health care workforce training programs (16–22). Most RE-AIM literature in health care looks at patient education programs instead. The present paper contributes to a growing body of information via this case study of the application of RE-AIM to a program evaluation effort for a PCP- and nurse-facing training intervention. A review of the literature found one paper (9) that updated and synthesized examples of uses of the RE-AIM framework and another (10) that summarized multiple interventions and concluded that RE-AIM can be applied to those which target professionals and operate at an individual, organizational, or community level. Another review (11) discussed changes to RE-AIM over the past 20 years as well as common misconceptions, and one paper (7) synthesized RE-AIM for pragmatic applications of the framework by health care topics targeted. However, none of these articles review literature on the use of RE-AIM for health care workforce training programs. More commonly, evaluators use other theories and frameworks to assess training programs, such as the Kirkpatrick model (12), which postulates four levels of training assessment outcomes: (1) Reaction, (2) Learning, (3) Behavior, and (4) Results. Other frameworks include Kaufman's or Anderson's Models of Learning Evaluation (13, 14). One recent paper (15) does look specifically at postgraduate rural medical training programs systematically, concluding with implications for rural workforce development, but it does not classify or organize by evaluation framework employed.

Among individual examples of RE-AIM used to assess training programs (16–22), evaluators turned to KAPS measures and participant intentions to reflect the Effectiveness of their programs. Programs defined RE-AIM components differently. For example, one project (21) that used train-the-trainer workshops to teach a tobacco cessation curriculum found that participants perceived that the workshop resulted in long-term, positive effects on their careers as well as their teaching and clinical practices. In another project (22), evaluators showed that an e-health COVID-19 educational intervention for health care workers resulted in participants' intentions to share the information and use it in clinic. Others also showed KAPS improvements for participants after attending lectures (18), or a training (20), or receiving educational material (17). However, each of these projects used different aspects of KAPS metrics to assess Effectiveness. Our experiences mirror the above. Given the variation in interpretation of RE-AIM components, the definitions that ORH's evaluation unit (24) provides to its funded programs offer a valuable element of conceptual standardization. This may help other projects, especially those focusing on the health care workforce.

In so far as limitations of the evaluation, we note that while the Rural WH-MR specified delineated goals, it did not set numerical objectives at the outset, which would have provided useful milestones and is recommended for similar future work. We mentioned above the lack of a comparison group, and the relatively small number of PCPs for whom we have measures both before and after the training, making KAPS changes more difficult to detect. We also found that although the program evaluation efforts thoroughly captured metrics for all RE-AIM components, this labor-intensive approach requires significant involvement from administrative, clinical, and evaluation partners to sustain it. Efforts to develop a master tracking database currently under construction will reduce the time needed to continue ongoing monitoring and automate aspects of program evaluation. In that regard, the program evaluation will more easily be maintained and sustained in the future.

In addition, we found the following limitations of the evaluation work hold. First, our solution to the COVID-19 pandemic delays complicated metrics assessment. For example, women Veterans on the panels of PCPs who partially completed the training but not fully, remain in the denominator of the R1 measure for our current analyses. This could lead to an underestimate of the program Reach because partially trained PCPs could be less inclined to increase the numbers of rural women Veterans on their panels than the fully trained PCPs. In other words, we may have a conservative estimate of program Reach. Second, after we developed our metrics, VA began its data migration to the Cerner Millennium information management system. While only one participating HCS migrated to Cerner during the training evaluation period, this change meant that some administrative data became unavailable via processes we relied upon. Third, measuring the degree to which providers and nurses engage in the training opportunity has proven challenging because the scheduling continues to be highly complex, with participants frequently replaced by their facilities due to changes in staff availability, interest, or need. On one hand, flexible scheduling allowed OWH to maximize training spaces but, on the other hand, it hindered our ability to definitively characterize the program Adoption at the PCP and nurse levels because the denominators represented a combination of participants either replaced or removed.

## Conclusion

Application of the RE-AIM framework proved valuable in the evaluation of the Rural WH-MR, demonstrating Effectiveness of this PCP- and nurse-facing training program. Our experience brought to light both pros and cons for RE-AIM's use to evaluate a workforce training program such as ours. On the positive side, the RE-AIM framework highlights both process and outcomes indicators of program successes and challenges. On the negative side, applying the framework to a provider- and nurse-facing intervention proved more complex. That is because, in the Rural WH-MR, PCPs and nurses represent a key target population of the intervention since the program seeks to improve their KAPS and comfort with women's health to improve outcomes for patients. However, in that regard, patients form a distal target population of the intervention as well. Once we decided to apply Reach to patients only and Adoption to PCPs/Nurses and HCSs/sites per ORH definitions, the framework demonstrated utility. RE-AIM enabled us to highlight all aspects of program development, execution, and evaluation, as well as generate some lessons learned—several of which have implications for similar workforce training programs in rural areas.

## Data Availability

The data presented in this paper represent non-research VA program evaluation data, which draws upon national VA databases. Patient medical record files from national VA databases cannot be released due to patient confidentiality. Investigators with regulatory permissions to use VA patient databases can access the raw patient data directly and can submit requests to the VA Office of Women's Health to access provider identifier codes that link to those databases, under a Memorandum of Understanding process. The algorithms used to pull those data, and analytic methods details, are available upon request to the authors.
