# Supplementary material for: RE-AIM applied to a primary care workforce training for rural providers and nurses: the Department of Veterans Affairs' Rural Women's Health Mini-Residency
Source: Front Health Serv. 2023 Nov 3;3:1205521. doi: 10.3389/frhs.2023.1205521 (PMC10656764; doi:10.3389/frhs.2023.1205521)
Supplement: Supplementary file 1 [file Table1.docx]

Supplementary Material

RE-AIM Applied to a Primary Care Workforce Training

For Rural Providers and Nurses:

The Department of Veterans Affairs’

Rural Women’s Health Mini-Residency

Rachel E. Golden DrPH, MPH^*^, Aimee M. Sanders MD, MPH, Susan M. Frayne MD, MPH

*Corresponding Author: Rachel.Golden@va.gov

**
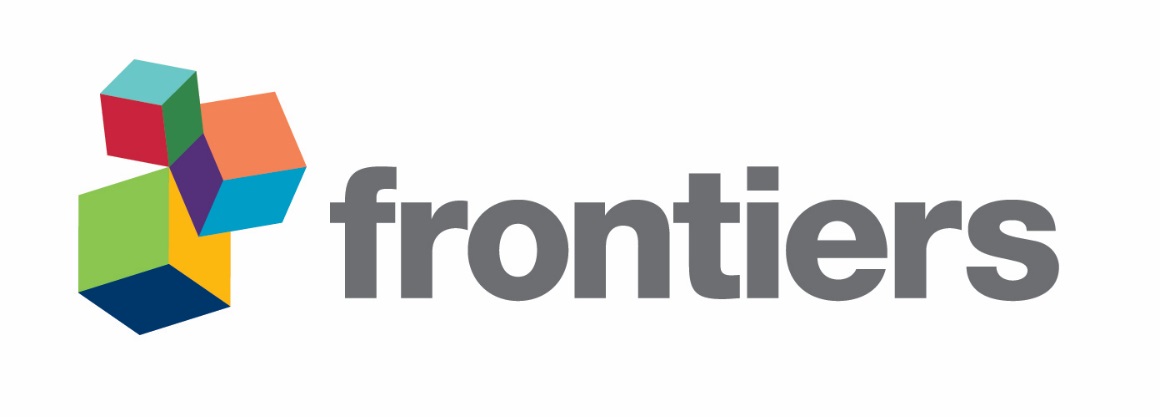
**

**Appendix A**

**Knowledge, Attitudes, Practices, and Skills (KAPS) Items**

**Assessed in the Rural Women’s Health Mini-Residency by Primary Care Provider and Nurses**

| **Primary Care Providers KAPS** |
| --- |
| **Overall, how comfortable are you in…?** |
| Caring for women Veterans |
| Discussing contraceptive options |
| Initiating contraception |
| Managing an abnormal Pap result |
| Evaluating vaginal discharge |
| Managing vaginal infections and sexually transmitted infections |
| Initiating the workup for abnormal uterine bleeding |
| Managing abnormal uterine bleeding |
| Initiating the workup for abdominal/pelvic pain |
| Managing abdominal/pelvic pain |
| Identifying & managing post-deployment issues specific to women Veterans |
| Managing menopausal symptoms |
| Identifying & managing intimate partner violence, Military Sexual Trauma and/or acute sexual assault |
| Identifying & initiating management for gynecologic emergencies such as ovarian torsion and ectopic pregnancy |
| Managing a breast mass |
| Performing a breast exam |
| Performing a pelvic exam |
| Specimen collection for Pap testing and vaginal discharge |
| Identifying normal & common abnormal pathologies of the breast & pelvis |
| Teaching trainees or colleagues about women's health topics |
| Identifying factors and tools that influence organizational change in women's health care |
| Working as part of a team to provide women’s health care |

| **Primary Care Nurses KAPS** |
| --- |
| **Overall, how comfortable are you in…?** |
| Caring for women Veterans |
| Describing advantages and disadvantages of contraceptive methods |
| Explaining Pap results and follow-up recommendations |
| Explaining the symptoms of sexually transmitted infections |
| History-taking or triaging for vaginal bleeding |
| History-taking or triaging for abdominal/pelvic pain |
| Providing nursing care for post-deployment issues specific to women Veterans |
| Discussing the clinical aspects of menopause |
| Providing nursing care to women who have experienced intimate partner violence, Military Sexual Trauma, and/or acute sexual assault |
| Discussing common causes of breast pain and various breast screening techniques |
| Providing nursing care before, during and after a breast exam |
| Providing nursing care before, during and after a pelvic exam |
| Identifying equipment and supply needs for various women's health exams and tests |
| Explaining gynecologic tests and procedures such as transvaginal ultrasound and endometrial biopsy |
| Discussing women's health topics with trainees or colleagues |
| Identifying factors and tools that influence organizational change related to women's health care |
| Working as part of a team to provide women's health care |
